# Remimazolam Versus Propofol for Anesthesia with I-Gel Airway Management During Coil Embolization of Unruptured Intracranial Aneurysms: A Randomized Controlled Trial

**DOI:** 10.3390/jcm15186986

**Published:** 2026-09-09

**Authors:** Juyeon Oh, Sung Yong Park, Ji Yeong Heo, Min Sun Park, Han Bum Joe

**Affiliations:** 1 Department of Anesthesiology and Pain Medicine, Asan Medical Center, University of Ulsan College of Medicine, Seoul 05505, Republic of Korea; itisamy@hanmail.net; 2 Department of Anesthesiology and Pain Medicine, Ajou University School of Medicine, Suwon 16499, Republic of Korea; anepark@aumc.ac.kr (S.Y.P.);

**Keywords:** anesthesia, coil embolization, hemodynamics, intracranial aneurysm, propofol, remimazolam, supraglottic airway

## Abstract

**Background**: Maintaining hemodynamic stability is crucial during coil embolization of unruptured intracranial aneurysms. Remimazolam has been suggested to exert less cardiovascular depression than propofol and may therefore provide more stable hemodynamic conditions. This study compared the hemodynamic effects of remimazolam and propofol when used for anesthesia induction and maintenance in patients undergoing coil embolization with I-gel airway management. **Methods**: In this single-center, randomized study, adults undergoing elective coil embolization for unruptured intracranial aneurysms were assigned to remimazolam or propofol induction followed by I-gel insertion. The primary outcome was the peak-to-nadir systolic blood pressure difference during the peri-induction period. Secondary outcomes included the peak-to-nadir mean arterial pressure difference, lowest systolic and mean arterial pressures, the incidence of hypotension, vasoactive drug requirements, and recovery outcomes. **Results**: Of the 50 patients randomized, 46 were included in the analysis, including 22 in the remimazolam group and 24 in the propofol group. The peri-induction peak-to-nadir systolic blood pressure difference was smaller with remimazolam than with propofol (38.2 ± 13.2 vs. 51.7 ± 13.4 mmHg, *p* = 0.001). The peak-to-nadir mean arterial pressure difference was also smaller, while the lowest systolic and mean arterial pressures were higher, with remimazolam. The incidence of hypotension was lower with remimazolam (40.9% vs. 87.5%, *p* = 0.002), with correspondingly lower requirements for ephedrine and norepinephrine. **Conclusions**: Remimazolam-based anesthesia was associated with a smaller peri-induction peak-to-nadir systolic blood pressure difference, a lower incidence of hypotension, and less frequent vasoactive drug use than propofol during I-gel-facilitated anesthesia for unruptured intracranial aneurysm coil embolization. These findings suggest a favorable hemodynamic profile in this setting and support further evaluation in larger trials.

## 1. Introduction

Endovascular coil embolization is an established treatment for unruptured intracranial aneurysms. General anesthesia is commonly used during this procedure to provide patient immobility, airway security, controlled ventilation, and stable procedural conditions [1,2]. Hemodynamic stability is also important because abrupt blood pressure changes may be undesirable in patients with intracranial aneurysms. Increases in arterial pressure may increase the transmural pressure gradient across the aneurysm wall, whereas excessive hypotension may reduce cerebral perfusion pressure [1,2,3]. Therefore, minimizing blood pressure fluctuation during anesthetic induction and airway manipulation is an important goal of anesthetic management in these patients.

Airway manipulation is a major contributor to hemodynamic responses during general anesthesia. Laryngoscopy and tracheal intubation can provoke cardiovascular and catecholamine responses [4], whereas supraglottic airway devices produce smaller hemodynamic responses than tracheal intubation [5]. I-gel, a second-generation supraglottic airway device, may further support this airway-sparing approach, as its insertion has been associated with more stable hemodynamic responses than endotracheal intubation [6]. However, reducing airway stimulation does not eliminate the risk of induction-related hypotension, particularly with propofol. In a previous study of propofol-based anesthesia with a laryngeal mask airway for endovascular treatment of unruptured intracranial aneurysms, mean arterial pressure and heart rate decreased significantly after anesthetic induction [3]. Thus, the choice of induction agent remains important in I-gel-based anesthesia.

Remimazolam is an ultra-short-acting benzodiazepine with a favorable cardiovascular profile and the additional advantage of flumazenil reversibility [7]. Recent studies in interventional neuroradiology and cerebrovascular surgery have suggested that remimazolam may reduce hypotensive events and vasoactive drug requirements compared with propofol- or volatile anesthetic-based regimens [8,9,10,11]. However, whether these hemodynamic advantages persist when airway-related sympathetic stimulation is already attenuated by the use of an I-gel supraglottic airway remains unclear. This question has particular clinical relevance because anesthetic induction and airway management occur before the aneurysm is secured, when both abrupt pressure increases and excessive hypotension are undesirable. Therefore, we conducted this randomized study to compare the hemodynamic effects of remimazolam and propofol in patients undergoing coil embolization with I-gel airway management, focusing on peri-induction blood pressure fluctuations, incidence of hypotension, and vasoactive drug requirements.

## 2. Materials and Methods

### 2.1. Study Design and Patients

This prospective, single-center, randomized controlled study was conducted at our tertiary hospital after approval by the institutional review board on 21 October 2022 (IRB No. AJOUIRB–IV–2022–417). The study protocol was submitted to the Clinical Research Information Service (CRIS) on 2 February 2023, and registered on 16 February 2023 (http://cris.nih.go.kr (accessed on 6 September 2026); KCT0008196). Written informed consent was obtained from all patients. Adults aged 20–65 years with American Society of Anesthesiologists physical status (ASA-PS) I–II who were scheduled for elective coil embolization of an unruptured intracranial aneurysm under general anesthesia were enrolled. Patients were excluded if they had preoperative systolic blood pressure (SBP) > 170 mmHg, body mass index > 30 kg/m^2^, anticipated difficult airway or difficult mask ventilation, chronic pulmonary disease, active respiratory infection, increased aspiration risk, allergy to benzodiazepines or opioids, history of drug abuse, pregnancy, breastfeeding, or refusal to participate. Eligible patients were randomly assigned in a 1:1 ratio to either the remimazolam or propofol group using a computer-generated random allocation sequence prepared by an independent investigator who was not involved in drug preparation, anesthesia management, vital sign recording, or outcome assessment. Study drugs were prepared according to group allocation by an anesthesia nurse who was not involved in randomization or outcome assessment. The patients and the investigator responsible for recording vital signs were blinded to group allocation. The attending anesthesiologist responsible for administering the study anesthetic was not blinded to group allocation because the two anesthetic regimens required different administration procedures and were clinically distinguishable. Intraoperative hemodynamic management was standardized according to prespecified criteria, as detailed below.

### 2.2. Anesthetic Protocol

No premedication was administered. Upon arrival in the neurointerventional suite, standard monitoring, including electrocardiography and pulse oximetry was applied. After local infiltration with 1% lidocaine, a 22-gauge angiocatheter was inserted into the radial artery for continuous blood pressure measurement. Anesthetic depth was monitored using a UniCon monitor (ADMS™, Anaesthetic Depth Monitoring System; Unimedics Co., Ltd., Seoul, Republic of Korea), which was attached to the patient’s forehead.

Patients were preoxygenated with 100% oxygen for 1 min. Before anesthetic induction, all patients received a standardized preload of balanced crystalloid solution at 5 mL/kg. Anesthesia was induced with an intravenous bolus of remimazolam 0.20 mg/kg in the remimazolam group and propofol 2.0 mg/kg in the propofol group [12,13,14,15,16,17]. Target-controlled infusion (TCI) of remifentanil using the Minto model was initiated at an effect-site concentration of 2.0 ng/mL [18]. After loss of consciousness, defined as the absence of response to verbal command, rocuronium 0.6 mg/kg was administered, and an I-gel (Intersurgical Ltd., Wokingham, UK) was inserted approximately 90–150 s after administration of the induction agent [19,20,21].

Following successful placement of the I-gel, the remifentanil target effect-site concentration was reduced to 1.0 ng/mL and was subsequently titrated according to individual clinical and hemodynamic responses. Anesthesia was maintained with a continuous infusion of remimazolam 1–2 mg/kg/h in the remimazolam group and propofol 5–10 mg/kg/h in the propofol group, with the maintenance dose of each agent titrated to keep the ADMS index within a range of 40–60 [15]. Continuous maintenance infusion of the assigned hypnotic agent was generally initiated approximately 10 min after I-gel insertion, guided by the patient’s hemodynamic status and ADMS index. Rocuronium was continuously infused at 0.2 mg/kg/h to maintain immobility throughout the intervention. Neuromuscular blockade was monitored using electromyography with a TetraSens^®^ electrode (Senzime AB, Uppsala, Sweden) connected to a TetraGraph monitor (Senzime AB, Uppsala, Sweden).

At the time of the last brain computed tomography scan after coil insertion, the infusions of remifentanil, rocuronium, and the assigned anesthetic agent—propofol or remimazolam—were discontinued. Neuromuscular blockade was antagonized using sugammadex (MSD, Seoul, Republic of Korea). In the remimazolam group, flumazenil 0.2 mg was administered at emergence. Recovery was assessed by the attending anesthesiologist. Time to eye opening was defined as the interval from discontinuation of the assigned maintenance anesthetic to eye opening in response to verbal command. The I-gel was removed after confirmation of adequate spontaneous ventilation, purposeful response to verbal command, and recovery of neuromuscular function, defined as a train-of-four ratio ≥ 0.9. The ADMS was selected for depth-of-anesthesia monitoring instead of the bispectral index monitor because the BIS forehead sensor contains metallic components that interfere with angiographic visualization. In contrast, the ADMS sensor does not contain metallic elements within the angiographic field, allowing continuous monitoring during coil embolization without obscuring the fluoroscopic images.

### 2.3. Hemodynamic Management and Data Collection

Hypotension was treated when SBP decreased by more than 30% from baseline or mean arterial pressure (MAP) was <65 mmHg. When hypotension was detected, intravenous ephedrine 4–8 mg was administered and could be repeated once if hypotension persisted or recurred. Persistent hypotension after the second dose prompted continuous norepinephrine infusion. Hypertension was defined as an increase in SBP of more than 30% from baseline or an SBP > 170 mmHg, and was treated with nicardipine 0.5–1 mg. Labetalol 2.5–5 mg was administered in cases of concomitant hypertension and tachycardia. Bradycardia, defined as a heart rate < 45 beats/min, was treated with intravenous atropine 0.5 mg, whereas tachycardia, defined as a heart rate > 100 beats/min, was treated with intravenous esmolol 5–10 mg. Blood pressure, heart rate, oxygen saturation, and anesthetic depth index were recorded at baseline, loss of consciousness, immediately after I-gel insertion, 5, 10, and 15 min after I-gel insertion, procedure start, angiography start, first coil deployment, discontinuation of maintenance anesthetics, and I-gel removal. Baseline and procedural variables collected for the present analysis included a history of hypertension, diabetes mellitus, dyslipidemia, aneurysm long- and short-axis sac diameters, neck diameter, aneurysm location, intraprocedural rupture or thromboembolic complications, and postprocedural complications. Perioperative data, including vasoactive drug use, anesthesia and procedure times, and time to eye opening, were also collected.

### 2.4. Outcomes

The primary outcome was the peak-to-nadir SBP difference during the peri-induction period, defined as the interval from pre-induction baseline to procedure start. For each patient, the peak-to-nadir difference was calculated as the highest minus the lowest value recorded during this period. The prespecified peri-induction time points used for this calculation were pre-induction baseline, loss of consciousness, immediately after I-gel insertion, 5, 10, and 15 min after I-gel insertion, and procedure start. Secondary hemodynamic outcomes included peak-to-nadir MAP difference and the lowest SBP and MAP during the peri-induction period, and the incidence of hypotension. The number of hypotensive episodes was additionally evaluated using 5 min blood pressure measurements, with consecutive hypotensive measurements considered a single episode until recovery above the predefined thresholds. Peak-to-nadir SBP and MAP differences during the overall anesthesia period (baseline to I-gel removal) were also evaluated. Additional secondary outcomes included vasoactive drug requirement, anesthetic depth index, and time to eye opening. Time-course changes in SBP from baseline to I-gel removal were also evaluated to describe overall hemodynamic trends during anesthesia.

### 2.5. Statistical Analysis

The analysis was performed using a modified intention-to-treat approach and included all randomized patients who received the allocated anesthetic intervention and had evaluable primary-outcome data. Continuous variables are presented as mean ± standard deviation, and categorical variables are presented as number (%). Between-group comparisons of continuous variables were performed using the independent *t*-test or the Mann–Whitney U test, based on the distributional characteristics of the data. Categorical variables were compared using the chi-squared test or Fisher’s exact test, as appropriate. The between-group distribution of the number of hypotensive episodes (0, 1, 2, or ≥3) was compared using the Fisher–Freeman–Halton exact test because of the small and sparse cell counts. For the principal continuous hemodynamic outcomes, effect sizes were calculated as Hedges’ g with 95% confidence intervals. Hedges’ g was calculated in the direction of remimazolam minus propofol. Time-course changes in systolic blood pressure and ADMS index were analyzed using two-way repeated-measures analysis of variance with group and time as factors; Greenhouse–Geisser correction was applied when the sphericity assumption was violated. For systolic blood pressure, between-group comparisons were performed at each of the 10 postinduction time points, with Bonferroni correction for multiple comparisons. Except for these Bonferroni-adjusted postinduction SBP time-point comparisons, secondary outcome analyses were not adjusted for multiplicity and should therefore be interpreted as exploratory. All tests were two-sided, and a *p* value < 0.05 was considered statistically significant. Statistical analyses were performed using IBM SPSS Statistics for Windows, version 32.0 (IBM Corp., Armonk, NY, USA).

### 2.6. Sample Size Calculation

The primary outcome of this study was the peak-to-nadir systolic blood pressure difference during the peri-induction period. Because no previous trial had directly compared this outcome between remimazolam and propofol—prior studies having compared the incidence of hypotension—a precise a priori calculation was not feasible. We therefore based the initial estimate on a previous study that compared the incidence of hypotension between the two agents [22], which yielded 54 patients per group. Because this initial estimate was derived from an indirect outcome, a separate pilot cohort was evaluated before initiation of the main randomized trial to obtain estimates for the primary outcome and refine the sample-size calculation. Fourteen patients, with 7 patients in each group, were evaluated separately from the main trial cohort. The primary hemodynamic endpoint for sample size re-estimation was the peak-to-nadir SBP difference during the peri-induction period, calculated as the highest minus the lowest SBP recorded between pre-induction baseline and procedure start. The actual pilot data used for sample-size re-estimation are provided in Supplementary Table S1. Based on the pilot-derived estimates from the anesthesia records, the mean ± SD peak-to-nadir SBP difference was 18.57 ± 7.81 mmHg in the remimazolam group and 42.29 ± 23.48 mmHg in the propofol group. These values corresponded to an anticipated treatment difference of −23.72 mmHg. The sample size was recalculated using PASS 14 Power Analysis and Sample Size Software (2015; NCSS, LLC, Kaysville, UT, USA) with a two-sided two-sample unequal-variance *t*-test, a significance level of 0.05, 90% power, and a 1:1 allocation ratio. This yielded 14 patients per group before adjustment for attrition. After accounting for a 10% dropout allowance and an additional 20% enrollment allowance for potential attrition, the required enrollment was calculated as 19.4 patients per group; therefore, at least 20 patients per group were considered necessary. To ensure enrollment above this minimum required sample size, 50 new patients were enrolled in the main trial.

## 3. Results

### 3.1. Patient Characteristics

**Figure 1:**
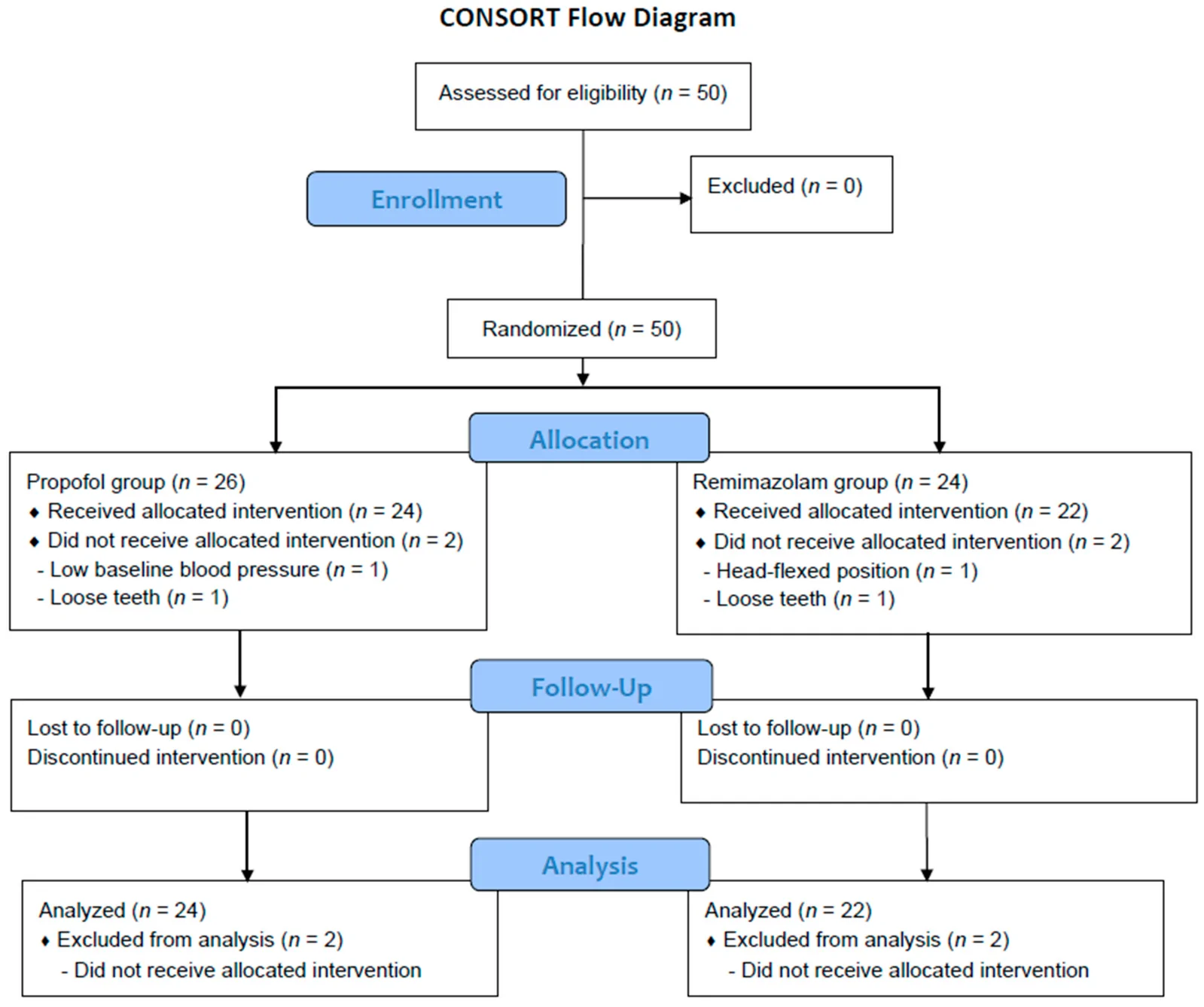
Consolidated Standards of Reporting Trials (CONSORT) flow diagram.

A total of 50 patients were enrolled and randomized. Four patients did not receive the allocated intervention and therefore had no evaluable primary-outcome data. These exclusions were identified during the final preinduction assessment in the neurointerventional suite before administration of the allocated anesthetic agent. The reasons were infeasibility of I-gel insertion in the required head-flexed position identified during preinduction airway and positioning assessment, without an actual I-gel insertion attempt (*n* = 1), unexpectedly low preinduction baseline blood pressure requiring norepinephrine before anesthetic induction despite acceptable preoperative screening blood pressure (*n* = 1), and the need for endotracheal intubation because of loose teeth identified during the final pre-airway examination (*n* = 2). Two patients were excluded from each group. Consequently, the final analysis included 46 patients, comprising 24 in the propofol group and 22 in the remimazolam group (Figure 1). Baseline patient and aneurysm characteristics are summarized in Table 1. No significant between-group differences were observed in demographic characteristics, comorbidities, aneurysm size or location, or baseline hemodynamic variables.

### 3.2. Hemodynamic Outcomes

Hemodynamic outcomes during the peri-induction and overall anesthesia periods are summarized in Table 2.

**Figure 2:**
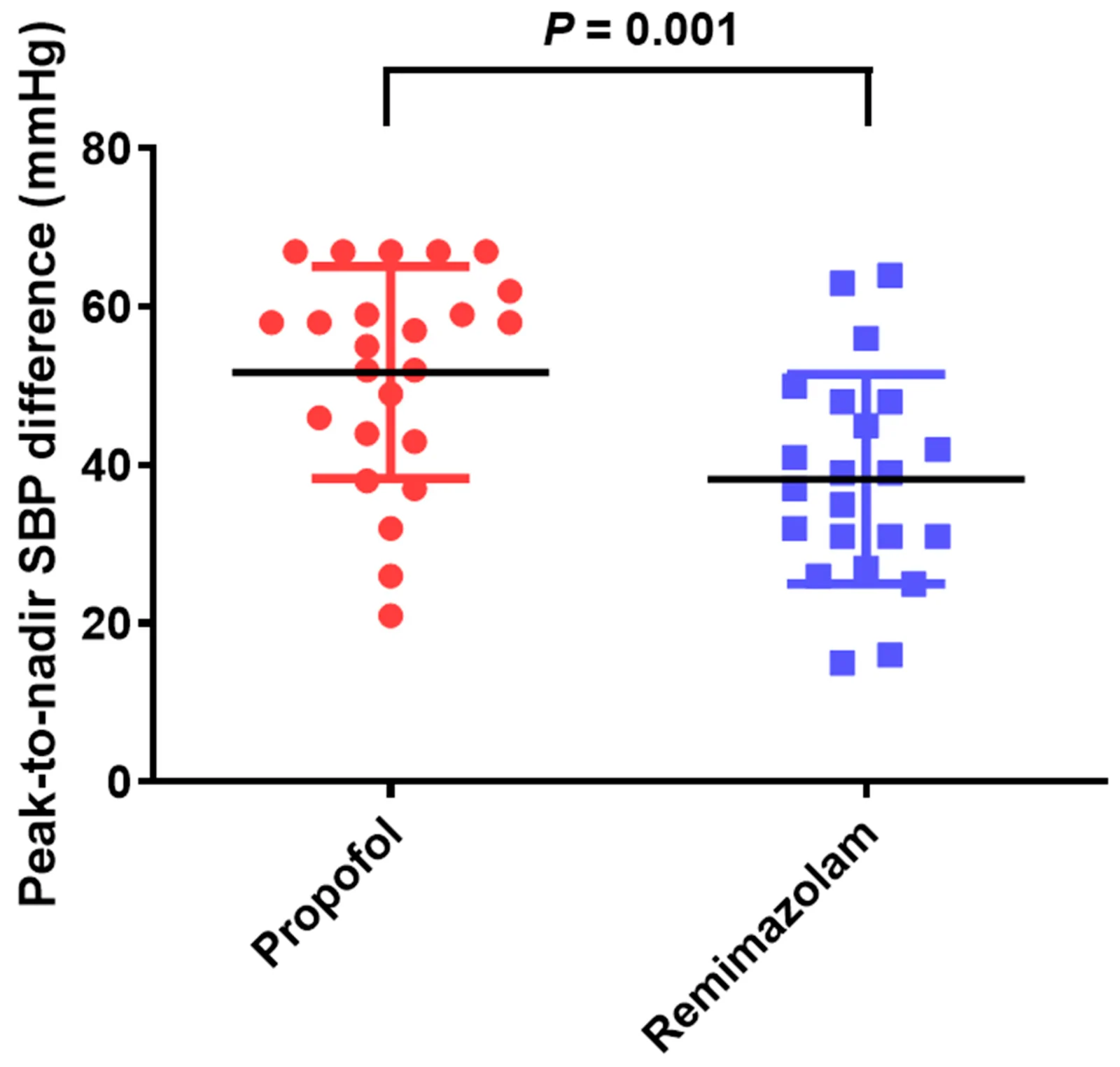
Peak-to-nadir systolic blood pressure difference during the peri-induction period. Individual data points show the peak-to-nadir systolic blood pressure difference for each patient during the peri-induction period, calculated as the difference between the highest and lowest systolic blood pressure values recorded during this period. Horizontal lines and error bars indicate mean ± SD. The peak-to-nadir systolic blood pressure difference was smaller in the remimazolam group than in the propofol group (38.2 ± 13.2 vs. 51.7 ± 13.4 mmHg, respectively).

For the primary outcome, the peri-induction peak-to-nadir SBP difference was smaller in the remimazolam group than in the propofol group (38.2 ± 13.2 vs. 51.7 ± 13.4 mmHg, *p* = 0.001) (Figure 2). The peri-induction peak-to-nadir MAP difference was also smaller in the remimazolam group (23.7 ± 7.9 vs. 33.5 ± 8.2 mmHg, *p* < 0.001), while the lowest SBP and MAP were higher (102.0 ± 14.8 vs. 90.3 ± 10.1 mmHg, *p* = 0.003; 66.2 ± 7.8 vs. 59.6 ± 7.7 mmHg, *p* = 0.006, respectively).

During the overall anesthesia period, the peak-to-nadir SBP and MAP differences remained smaller in the remimazolam group than in the propofol group (SBP, 42.9 ± 11.7 vs. 53.9 ± 12.0 mmHg, *p* = 0.003; MAP, 29.0 ± 8.4 vs. 36.4 ± 7.7 mmHg, *p* = 0.003).

### 3.3. Time-Course Changes in Systolic Blood Pressure

**Figure 3:**
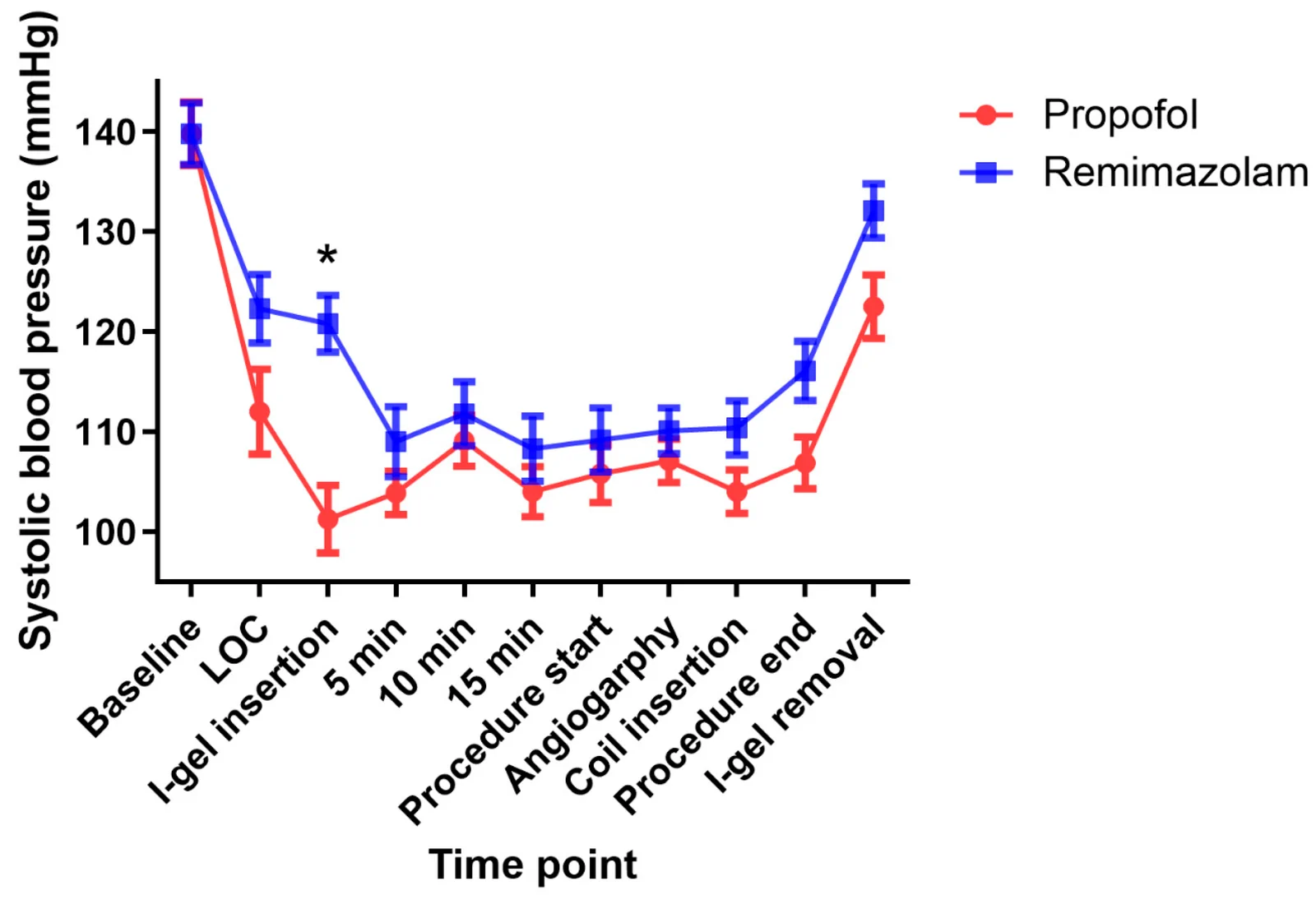
Time-course changes in systolic blood pressure during anesthesia. Systolic blood pressure values at each time point in the propofol and remimazolam groups. Values are presented as mean ± standard error. Baseline, before anesthetic induction; LOC, loss of consciousness; I-gel insertion, immediately after I-gel insertion; 5, 10, and 15 min after I-gel insertion; Procedure start; Angiography; Coil insertion, first coil deployment; Procedure end, discontinuation of maintenance anesthetics; I-gel removal. * *p* < 0.05 versus propofol after Bonferroni correction across the 10 postinduction time-point comparisons.

Time-course changes in SBP are shown in Figure 3. In both groups, SBP decreased after anesthetic induction. Repeated-measures analysis of variance with Greenhouse–Geisser correction showed a significant group-by-time interaction (*p* = 0.019), indicating that the temporal pattern of SBP change differed between the two groups. In Bonferroni-adjusted comparisons across the 10 postinduction time points, SBP was significantly higher in the remimazolam group than in the propofol group only immediately after I-gel insertion (adjusted *p* < 0.001), whereas no significant between-group differences were observed at the other time points.

### 3.4. Hypotension, Vasoactive Drug Requirement, Anesthetic Depth, and Recovery Outcomes

Hypotension, vasoactive drug requirement, procedural, and recovery outcomes are summarized in Table 3. Hypotension occurred less frequently in the remimazolam group than in the propofol group (40.9% vs. 87.5%, *p* = 0.002). The number of hypotensive episodes was also lower in the remimazolam group (0/1/2/≥3 episodes: 13/4/2/3 vs. 3/6/5/10, *p* = 0.007). Ephedrine was used less frequently in the remimazolam group than in the propofol group (31.8% vs. 79.2%, *p* = 0.001). Norepinephrine use was also less frequent in the remimazolam group (4.5% vs. 41.7%, *p* = 0.003). Among patients receiving chronic antihypertensive medications, these medications were continued on the day of the procedure and were not withheld. Regarding other recorded safety variables, hypertension requiring nicardipine occurred in one patient in the remimazolam group. Neither group experienced bradycardia requiring atropine, oxygen desaturation, or airway-related adverse events. Transient tachycardia was observed in two patients but did not require esmolol. No intraprocedural rupture or thromboembolic complication occurred in either group. One patient in the propofol group experienced a transient ischemic attack during hospitalization, whereas no postprocedural complication occurred in the remimazolam group.

**Figure 4:**
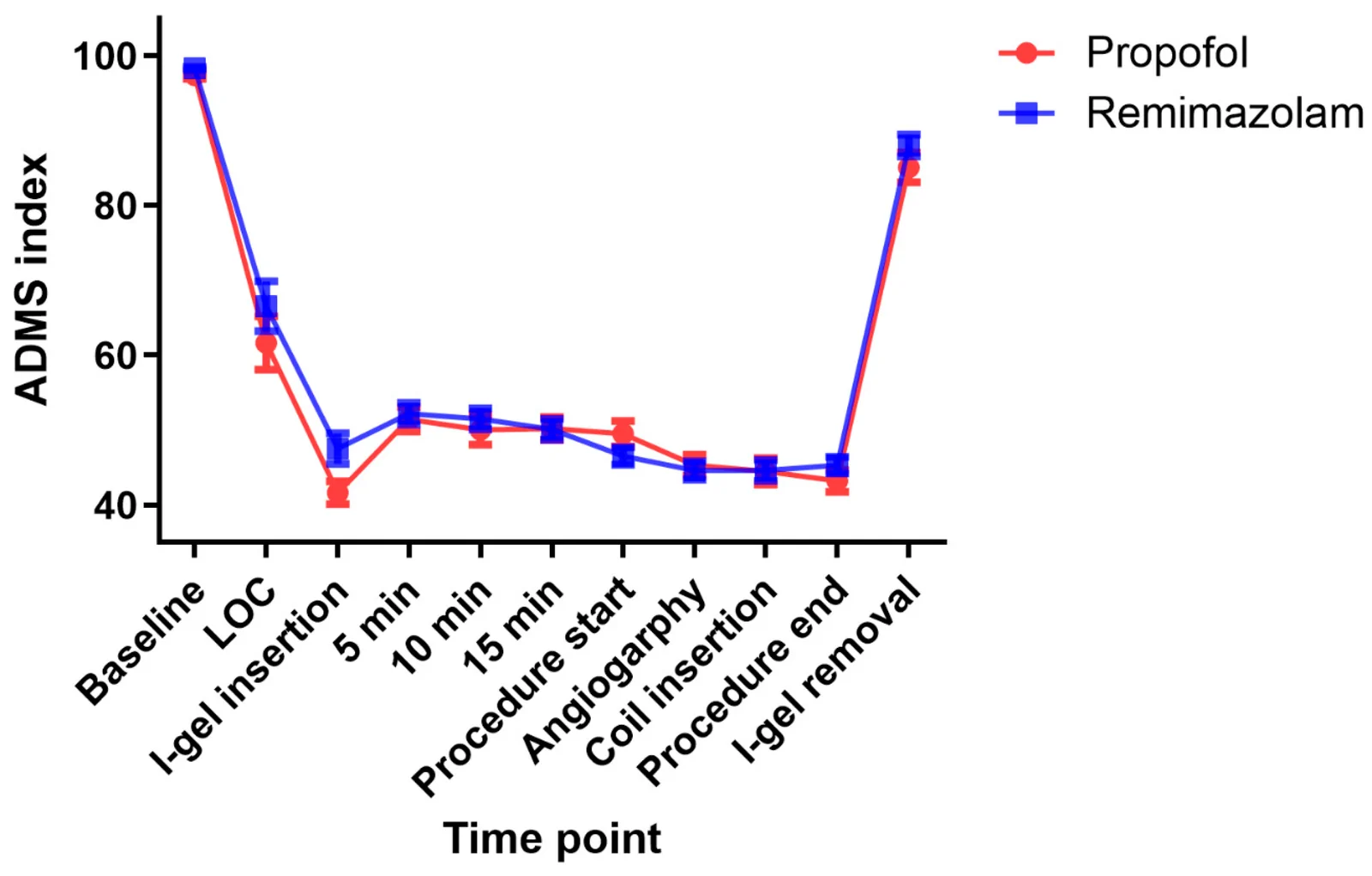
Time-course changes in the anesthetic depth monitoring system (ADMS) index during anesthesia. ADMS index values at each time point in the propofol and remimazolam groups. Values are presented as mean ± standard error. Baseline, before anesthetic induction; LOC, loss of consciousness; I-gel insertion, immediately after I-gel insertion; 5, 10, and 15 min after I-gel insertion; Procedure start; Angiography; Coil insertion, first coil deployment; Procedure end, discontinuation of maintenance anesthetics; I-gel removal.

Time-course changes in the ADMS index are shown in Figure 4. ADMS values at loss of consciousness were 61.6 ± 17.5 in the propofol group and 66.5 ± 15.6 in the remimazolam group; the corresponding values immediately after I-gel insertion were 41.6 ± 7.6 and 47.5 ± 9.5, respectively. Repeated-measures analysis of variance with Greenhouse–Geisser correction showed no significant group-by-time interaction (*p* = 0.304), indicating that the temporal pattern of ADMS index did not differ significantly between the two groups.

Time to eye opening was shorter in the remimazolam group than in the propofol group (4.8 ± 2.9 vs. 6.6 ± 2.8 min, *p* = 0.019).

## 4. Discussion

In this single-center randomized study of patients undergoing coil embolization for unruptured intracranial aneurysms with I-gel airway management, remimazolam-based anesthesia was associated with a smaller peak-to-nadir systolic and mean arterial pressure differences during the peri-induction period, higher lowest systolic and mean arterial pressures, a lower incidence of hypotension, and less frequent use of ephedrine and norepinephrine than propofol-based anesthesia. Peak-to-nadir systolic and mean arterial pressure differences were also smaller with remimazolam over the overall anesthesia period. Together, these findings suggest that remimazolam may complement an I-gel-based airway-sparing strategy by providing a more favorable hemodynamic profile with a lower requirement for pharmacological support.

Hemodynamic stability is an important goal during anesthetic management for unruptured intracranial aneurysm coil embolization. Cerebral perfusion pressure is determined by the difference between mean arterial pressure and intracranial pressure; therefore, when intracranial pressure remains stable, a reduction in mean arterial pressure directly decreases cerebral perfusion pressure. Although cerebral autoregulation normally maintains cerebral blood flow across a range of arterial pressures, excessive hypotension may compromise cerebral blood flow if arterial pressure falls below the lower limit of autoregulation [2,23]. Conversely, abrupt hypertension may increase the transmural pressure gradient across the aneurysm wall and the risk of rupture before the aneurysm is completely secured [2,24]. Maintaining blood pressure within a narrow and stable range is therefore particularly important during anesthetic induction and airway management, when the cardiovascular depressant effects of anesthetic agents and the sympathetic response to airway manipulation may produce rapid hemodynamic changes [1,25]. In the present study, compared with propofol, remimazolam was associated with a smaller peri-induction peak-to-nadir systolic blood pressure difference (Hedges’ g, −1.00, 95% CI, −1.60 to −0.39, *p* = 0.001). The narrower systolic blood pressure excursion may be clinically relevant because minimizing abrupt pressure shifts before the aneurysm is completely secured may limit sudden changes in the transmural pressure gradient across the aneurysm wall. At the same time, the lower incidence of hypotension and less frequent vasoactive drug use further support a more stable hemodynamic profile with remimazolam during induction.

Recent randomized studies have reported favorable hemodynamic profiles of remimazolam in neurovascular anesthesia. Lee et al. found that mean arterial pressure was higher with remimazolam than with propofol at several early time points after anesthetic induction during coil embolization, although the overall mean arterial pressure trajectory did not differ significantly between groups [8]. Similarly, Koo et al. reported less frequent and shorter episodes of hypotension, higher lowest mean arterial pressure, and lower blood pressure variability with remimazolam during both the induction and maintenance phases of cerebrovascular bypass surgery, although the comparator regimen consisted of propofol induction followed by desflurane maintenance [11]. Our results are consistent with these findings and further characterize the peri-induction hemodynamic profile of remimazolam. Unlike the mixed intravenous–volatile regimen used in the control group by Koo et al., the assigned anesthetic was used for both induction and maintenance in our study, allowing a more direct comparison of remimazolam- and propofol-based anesthetic strategies. More recently, Duan et al. reported lower intraoperative mean arterial pressure variability with remimazolam than with propofol during endovascular embolization of intracranial aneurysms, based on average real variability and other variability indices [26]. Their study primarily characterized blood pressure variability throughout the intraoperative period. In our study, we evaluated the peak-to-nadir systolic and mean arterial pressure differences during the peri-induction period and across the overall anesthesia period. These complementary measures suggest that remimazolam attenuated blood pressure fluctuations during induction and throughout anesthesia. The clinical significance of the present study lies in its focus on the peri-induction interval before the aneurysm is secured, when both abrupt pressure increases and excessive hypotension are undesirable. The observed differences in blood pressure fluctuation and vasoactive drug requirements within an I-gel-based, relatively low-stimulation anesthetic strategy suggest that anesthetic selection may remain clinically relevant even when airway-related stimulation is minimized.

The lower requirement for vasoactive support provides an important complement to the measured blood pressure data. Although arterial pressures became similar during the maintenance period, this convergence was achieved with substantially more rescue treatment in the propofol group. Ephedrine was required in 79.2% of patients receiving propofol compared with 31.8% of those receiving remimazolam, while norepinephrine was required in 41.7% and 4.5%, respectively. Because norepinephrine infusion was initiated only when hypotension persisted despite ephedrine administration, its lower use in the remimazolam group indicates a less frequent need for escalation of pharmacological support. Similar reductions in vasoactive drug requirements with remimazolam have been reported during interventional neuroradiology and cerebrovascular bypass surgery [8,11]. These findings suggest that remimazolam allowed stable procedural blood pressure to be maintained with a lower hemodynamic treatment burden. This reduction in vasoactive support may be particularly relevant in neurointerventional anesthesia. Although vasoactive drugs can restore systemic arterial pressure during hypotension, their effects on cerebral perfusion and oxygenation are not uniform and may depend on factors such as cardiac output, baseline blood pressure, cerebral autoregulatory status, and the magnitude of the pressure increase [27,28,29]. Therefore, the reduced requirement for vasoactive support may represent a favorable hemodynamic feature of remimazolam in low-stimulation neurointerventional procedures. However, because cerebral perfusion, cerebral oxygenation, and standardized neurological outcomes were not assessed, these findings should not be interpreted as evidence of neurophysiological or neurological benefit.

Recovery is relevant in neurointerventional anesthesia because early postprocedural neurological assessment may facilitate the detection of procedure-related complications [1,2,24]. In the present study, the time to eye opening was significantly shorter in the remimazolam group than in the propofol group (4.8 ± 2.9 vs. 6.6 ± 2.8 min, *p* = 0.019). However, the absolute difference was modest, and its clinical significance remains uncertain. Because flumazenil was administered only in the remimazolam group, this finding should be interpreted as reflecting a remimazolam–flumazenil strategy rather than the intrinsic recovery profile of remimazolam alone. This pharmacological reversibility may be advantageous in neurointerventional anesthesia by allowing earlier restoration of a neurologically assessable state; nevertheless, direct comparison of recovery profiles between remimazolam and propofol should be interpreted cautiously.

This study has several limitations. First, this was a single-center study with a relatively small sample size, and the sample-size calculation was based on a separate pilot cohort of seven patients per group. Although the main trial met the prospectively determined enrollment target, the limited pilot sample may have resulted in imprecise estimates of the treatment effect and variance. Therefore, the magnitude of the observed treatment effect should be interpreted cautiously, and the present findings should be regarded as exploratory pending confirmation in larger multicenter trials. Second, although arterial blood pressure was continuously monitored for clinical care, the primary outcome was calculated from prespecified peri-induction time-point recordings rather than continuous waveform extraction. Therefore, the true nadir SBP may not have been captured if it occurred between scheduled recording points, and the calculated peak-to-nadir SBP difference may have underestimated the actual maximum blood pressure excursion in some patients. This limitation is particularly relevant because the primary outcome directly depended on identifying the nadir SBP. Nevertheless, the same prespecified recording schedule was applied to both groups. Third, four randomized patients who did not receive the allocated intervention were excluded from the analysis. Although the exclusions occurred before administration of the study anesthetic and were balanced between groups, post-randomization exclusion may have reduced the protection afforded by randomization and introduced potential bias. Fourth, the attending anesthesiologist could not be blinded to group allocation. Although intraoperative hemodynamic management was standardized according to prespecified criteria, potential performance bias cannot be entirely excluded. Fifth, because fixed induction doses were used, equivalent anesthetic depth throughout induction could not be ensured. Although the overall temporal pattern of the ADMS index did not differ significantly between groups, the potential influence of anesthetic depth should be considered when interpreting the hemodynamic findings. Sixth, because the study focused on patients undergoing coil embolization for unruptured intracranial aneurysms with I-gel airway management, the findings may not be directly applicable to procedures requiring tracheal intubation, ruptured aneurysm treatment, or more highly stimulating neurosurgical procedures. Seventh, this study primarily evaluated hemodynamic outcomes and vasoactive drug requirements without direct assessment of cerebral perfusion, cerebral oxygenation, or postoperative neurological outcomes. Therefore, although remimazolam was associated with more stable blood pressure, whether these hemodynamic benefits translate into improved neurophysiological or clinical outcomes remains uncertain.

In conclusion, remimazolam was associated with a smaller peri-induction peak-to-nadir systolic blood pressure difference, a lower incidence of hypotension, and less frequent use of ephedrine and norepinephrine than propofol in patients undergoing coil embolization for unruptured intracranial aneurysms with I-gel airway management. These findings characterize the peri-induction hemodynamic profile of remimazolam during the clinically critical period before the aneurysm is secured, when minimizing both hypotension and abrupt blood pressure changes is particularly important. However, the precision and generalizability of these findings remain limited, and larger multicenter trials are needed for confirmation.

## Figures and Tables

**Table 1:** Baseline patient and aneurysm characteristics.

Variable	Propofol (*n* = 24)	Remimazolam (*n* = 22)	*p*-Value
Age (years)	53.7 ± 6.4	54.3 ± 4.5	0.735
Sex (M/F)	7/17	11/11	0.148
Weight (kg)	62.6 ± 11.8	67.8 ± 10.8	0.121
Height (cm)	161.3 ± 8.3	164.6 ± 9.9	0.205
BMI (kg/m^2^)	23.9 ± 3.2	24.9 ± 2.6	0.250
ASA-PS, *n* (%)			0.250
I	6 (25.0)	9 (40.9)	
II	18 (75.0)	13 (59.1)	
Comorbidities, *n* (%)			
Hypertension	9 (37.5)	13 (59.1)	0.143
Diabetes mellitus	5 (20.8)	4 (18.2)	1.000
Dyslipidemia	6 (25.0)	5 (22.7)	0.857
Aneurysm size, mm			
Long-axis sac diameter	5.33 ± 1.68	4.82 ± 1.75	0.156
Short-axis sac diameter	4.14 ± 1.24	3.67 ± 0.99	0.222
Neck diameter	3.43 ± 1.07	3.57 ± 1.21	0.930
Aneurysm location, *n* (%)			0.791
ICA paraclinoid	11 (45.8)	8 (36.4)	
Acom	3 (12.5)	5 (22.7)	
Pcom	4 (16.7)	3 (13.6)	
Other	6 (25.0)	6 (27.3)	
Baseline hemodynamic variables			
Baseline SBP, mmHg	139.8 ± 15.6	139.8 ± 14.5	0.989
Baseline MAP, mmHg	91.0 ± 11.4	89.3 ± 8.6	0.577
Baseline heart rate, beats/min	77.8 ± 13.0	74.7 ± 13.1	0.417

Values are presented as mean ± SD or number (%). Other aneurysm locations included ICA and MCA bifurcations, ICA ophthalmic artery, ICA cavernous segment, ICA supraclinoid segment, ICA dorsal wall, and basilar tip. BMI: body mass index, ASA-PS: American Society of Anesthesiologists physical status, ICA: internal carotid artery, MCA: middle cerebral artery, Acom: anterior communicating artery, Pcom: posterior communicating artery, SBP: systolic blood pressure, MAP: mean arterial pressure.

**Table 2:** Hemodynamic outcomes during the peri-induction and overall anesthesia periods.

Outcome	Propofol (*n* = 24)	Remimazolam (*n* = 22)	Mean Difference (95% CI)	Effect Size (95% CI)	*p*-Value
	Peri-induction period (baseline to procedure start)				
Peak-to-nadir SBP difference, mmHg	51.7 ± 13.4	38.2 ± 13.2	−13.5 (−21.4 to −5.6)	−1.00 (−1.60 to −0.39)	0.001
Peak-to-nadir MAP difference, mmHg	33.5 ± 8.2	23.7 ± 7.9	−9.9 (−14.6 to −5.1)	−1.21 (−1.82 to −0.58)	<0.001
Lowest SBP, mmHg	90.3 ± 10.1	102.0 ± 14.8	11.7 (4.1 to 19.3)	0.92 (0.32 to 1.52)	0.003
Lowest MAP, mmHg	59.6 ± 7.7	66.2 ± 7.8	6.6 (1.9 to 11.2)	0.83 (0.23 to 1.42)	0.006
	Overall anesthesia period (baseline to I-gel removal)				
Peak-to-nadir SBP difference, mmHg	53.9 ± 12.0	42.9 ± 11.7	−11.2 (−18.4 to −3.9)	−0.91 (−1.50 to −0.31)	0.003
Peak-to-nadir MAP difference, mmHg	36.4 ± 7.7	29.0 ± 8.4	−7.4 (−12.2 to −2.6)	−0.90 (−1.50 to −0.30)	0.003

Values are presented as mean ± SD. Mean differences were calculated in the direction of remimazolam minus propofol and are presented with 95% confidence intervals. Effect sizes are presented as Hedges’ g with 95% confidence intervals and were calculated in the direction of remimazolam minus propofol. CI: confidence interval, SBP: systolic blood pressure, MAP: mean arterial pressure.

**Table 3:** Hypotension, vasoactive drug requirements, anesthetic, procedural and recovery outcomes.

Outcome	Propofol (*n* = 24)	Remimazolam (*n* = 22)	Risk Ratio (95% CI)	*p*-Value
Incidence of hypotension, *n* (%)	21 (87.5)	9 (40.9)	0.47 (0.28 to 0.79)	0.002
Number of hypotensive episodes, *n* (0/1/2/≥3)	3/6/5/10	13/4/2/3		0.007
Ephedrine use, *n* (%)	19 (79.2)	7 (31.8)	0.40 (0.21 to 0.76)	0.001
Ephedrine dose among users (mg)	10.6 ± 4.1	6.6 ± 1.5		-
Norepinephrine use, *n* (%)	10 (41.7)	1 (4.5)	0.11 (0.02 to 0.78)	0.003
Norepinephrine dose among users, μg	64.7 ± 26.4	75.0		-
Remifentanil total dose, μg	280.5 ± 75.1	307.3 ± 120.2		0.364
Procedure time, min	40.8 ± 10.1	45.7 ± 15.5		0.362
Anesthesia time, min	86.0 ± 13.1	88.9 ± 18.4		0.550
Intraoperative fluid administration, mL	755.4 ± 171.3	834.1 ± 180.9		0.082
Intraoperative urine ouput, mL	244.2 ± 147.7	287.7 ± 206.2		0.412
Intraprocedural rupture or thromboembolism, *n*	0	0		-
Postprocedural complication, *n* (%)	1 (4.2)	0 (0)		1.000
Time to eye opening, min	6.6 ± 2.8	4.8 ± 2.9		0.019

Values are presented as mean ± SD or number (%). Hypotension was defined as a decrease in SBP of more than 30% from baseline or MAP < 65 mmHg. Risk ratios were calculated for the incidence of hypotension and binary vasoactive-drug use outcomes in the direction of remimazolam versus propofol and are presented with 95% confidence intervals. For the number of hypotensive episodes, values indicate the numbers of patients with 0, 1, 2, or ≥3 episodes, respectively. The *p* value for the number of hypotensive episodes was obtained using the two-sided Fisher–Freeman–Halton exact test. Doses of vasoactive drugs were summarized only among patients who received the corresponding drug and were not statistically compared because of the small and imbalanced number of users. The norepinephrine dose in the remimazolam group represents the value for a single patient. The only postprocedural complication was a transient ischemic attack in the propofol group. CI: confidence interval, SBP: systolic blood pressure, MAP: mean arterial pressure.

## Data Availability

The data presented in this study are available upon request from the corresponding author.

## Supplementary Materials

The following supporting information can be downloaded at: https://www.mdpi.com/article/10.3390/jcm15186986/s1. Table S1: Pilot data used for sample-size re-estimation.
