# Supplementary material for: Remimazolam Versus Propofol for Anesthesia with I-Gel Airway Management During Coil Embolization of Unruptured Intracranial Aneurysms: A Randomized Controlled Trial
Source: J Clin Med. 2026 Sep 9;15(18):6986. doi: 10.3390/jcm15186986 (PMC13607978; doi:10.3390/jcm15186986)
Supplement: Supplementary file 1 [file jcm-15-06986-s001.zip › jcm-4539717-supplementary.pdf]

Journal of
Clinical Medicine

MDPI

Supplementary Table S1. Pilot data used for sample-size re-estimation.

Group	Highest SBP during peri-induction period, mmHg	Lowest SBP during peri-induction period, mmHg
Remimazolam	148	132
Remimazolam	152	132
Remimazolam	156	146
Propofol	152	88
Remimazolam	128	116
Remimazolam	150	136
Propofol	140	122
Propofol	158	96
Remimazolam	118	88
Propofol	152	148
Propofol	140	102
Propofol	152	102
Propofol	146	86
Remimazolam	120	92

Values are individual patient data from the separate pilot cohort (n = 14; 7 patients per group). The peri-induction period was defined as the interval from the pre-induction baseline to procedure start. The peak-to-nadir SBP difference was calculated for each patient as the highest minus the lowest SBP recorded during this period. The pilot cohort was not included in the main randomized trial and was used only for sample-size re-estimation. SBP: systolic blood pressure.
